# The Adipokine Profile and the Cardiometabolic Risk in Non-Obese Young Adults

**DOI:** 10.4274/balkanmedj.galenos.2018.2018.0789

**Published:** 2019-05-10

**Authors:** Marina Ruxandra Otelea, Adrian Streinu-Cercel, Cristian Băicus, Maria Nitescu

**Affiliations:** 1 Carol Davila University of Medicine and Pharmacy, Bucharest, Romania; 2 Institute for Infectious Diseases ‘Matei Bals’, Bucharest, Romania; 3 Clinical Hospital Colentina, Bucharest, Romania

**Keywords:** Adipokines, cardiovascular disease, insulin resistance, non-obese, young adult

## Abstract

**Background:**

Young, non-obese adults are considered at low risk for cardiometabolic diseases, although markers of an unhealthy metabolic state are not uncommon findings in this population. Adipose tissue dysfunction, evaluated by the adipokine profile, significantly influences lipid and glucose metabolism and low-grade systemic inflammation.

**Aims:**

To determine the relation between adipose tissue dysfunction and the already confirmed cardiometabolic risk indicators, including the atherogenic index of plasma, lipid accumulation product, homeostatic model assessment of insulin resistance, and the low-grade inflammation markers, namely, interleukin 6 and high-sensitivity C-reactive protein.

**Study Design:**

Cross-sectional study.

**Methods:**

We recruited 93 non-obese, healthy young adults. Anthropometric, lipid profile, inflammatory markers, and adipokines were measured. An abnormal adipokine profile (high leptin-to-adiponectin ratio) was considered as a marker of a dysfunctional adipose tissue. The correlation between the leptin-to-adiponectin ratio and the anthropometric measurements, atherogenic index of plasma, lipid accumulation product, homeostatic model assessment of insulin resistance, interleukin 6, and high-sensitivity C-reactive protein was determined.

**Results:**

We found a direct correlation between the abnormal adipokine profile and the cardiometabolic risk indicators mentioned above, except for the low-grade inflammatory markers. In the regression model derived from our data, the leptin-to-adiponectin ratio was best correlated with the unfavorable plasma lipid profile, as estimated by the atherogenic index of plasma (r=0.097, confidence interval=0.015-0.180, p=0.021). A significantly higher leptin-to-adiponectin ratio was found in the insulin-resistant group (p=0.012) and in the highest lipid accumulation product quartile (p=0.032).

**Conclusion:**

In a non-obese young population, the high rate of leptin-adiponectin may be a good predictor of cardiovascular and metabolic risk assessment.

The discovery of adiponectin and its anti-inflammatory and vasoprotective actions was enthusiastically expected to solve the link between obesity and cardiovascular diseases. Although the majority of the adiponectin pool is produced by the mature, fully differentiated adipocytes (1), serum levels tend to decrease in the obese population. In contrast, the leptin levels increase, contributing to the pro-inflammatory status of obesity. These findings support the hypothesis that, up from a certain point, an increase in fat mass changes the normal adipokine profile of the adipocytes and triggers metabolic dysfunction (2). Consequently, the unfavorable adipokine profile was added to the characteristics of the unhealthy fat tissue definition apart from the specific neurohormonal response, the predominant visceral localization, and the inflammatory infiltration orchestrated by the macrophage M1 subtype (3). The unhealthy transformation of the adipose tissue could provide the physiopathological explanation for both the non-obese metabolically obese phenotype and the obese non-metabolically obese individuals. The non-obese metabolically obese individuals have a body mass index (BMI) of <30 in which multiple components of the metabolic syndrome are present, with a high ratio between the fat mass and the free-fat mass and a predominant abdominal fat disposition (3). Methods to determine these characteristics are not yet available for screening purposes, except for the waist circumference, a surrogate for the visceral fat assessment. The fact that not all obese persons have predominantly unhealthy adipose tissue could also explain the percentage of obese non-metabolically obese subjects.

Risk models based on lipid profile and inflammatory markers are already included in the cardiovascular guidelines, but under the current recommendations, the young, non-obese population is considered at low risk. In this study, we tested the hypothesis of an existing relationship between the adipose tissue dysfunction, estimated by the leptin-to-adiponectin ratio (LAR), and the vascular and metabolic risk indicators in this particular population.

The primary objective of our study was to identify the relation between the LAR and the atherogenic index of plasma (AIP), a synthetic indicator of a disturbed lipid metabolism (4).

## MATERIALS AND METHODS

We recruited young, normal-weight, and healthy individuals. Therefore, the study was opened to the local community of medical students. The participation was voluntary with no positive selection criteria for enrollment. Besides a preexisting chronic disease, the exclusions referred to any already diagnosed medical condition that could affect the lipid metabolism or would have been related to the metabolic syndrome. The exclusion criteria for enrollment were as follows: any preexisting chronic medical conditions, current blood pressure medication use, prediagnosed diabetes or metabolic syndrome, polycystic ovarian syndrome, and hypothyroidism. The study was approved by the Ethical Committee of the "Matei Bals" National Institute of Infectious Diseases. Written informed consent was obtained from all individuals before study commencement.

After applying the exclusion criteria related to the preexisting medical conditions, 93 subjects (30 men and 63 women) completed the full protocol. The questionnaire included basic information on smoking (smoker or nonsmoker) and alcohol consumption (average drink per day). The physical activity questionnaire included a list of the most popular recreational activities in the community, their frequency, and duration. Waist circumference was measured at the midway between the lower margin of the last rib and the iliac crest, without compressing the soft tissues. Blood pressure value represents the mean of three measurements assessed in a supine position after 5 min of rest. Blood samples were collected in the morning, after a minimum of 10 h of fasting. The spectrophotometric method was used for cholesterol fractions, the Jaffe method for creatinine, and ELISA tests for insulin (Ins-ELISA KAP1251, DIAsource), leptin (Leptin ELISA KAP2281, DIAsource), and adiponectin (Adiponectin ELISA KAPME09, DIAsource). The liver cytolysis was estimated from plasma transaminases. Chronic viral hepatitis was excluded from the screening tests. The lipid blood profile included triglycerides, high-density lipoprotein cholesterol, and low-density lipoprotein cholesterol. Normal values for the definition of the metabolic syndrome refer to thresholds indicated by the International Diabetes Federation for Caucasians (5). For the other laboratory tests performed, the normal reference of the test as indicated by the kit manufacturer was used.

The AIP was calculated based on the formula: AIP=log (triglycerides/high-density lipoprotein cholesterol) (6). We stratified our population according to the AIP value reported in the previous studies (4). The subjects were divided into three groups, those with low risk (AIP <0.11), medium risk (AIP 0.11-0.21), and high risk (AIP >0.21).

The LAP was calculated as [waist circumference (cm) - 65] × [triglycerides concentration (mM)] for men and [waist circumference (cm) - 58] × [triglycerides concentration (mM)] for women. The highest quartile cut-off was considered for comparison (7). In our sample, this value was 48.28 for women and 51.77 for men.

In regard to the adipose tissue secretion profile, we measured adiponectin and leptin and calculated the LAR.

Homeostatic model assessment of insulin resistance (HOMA-IR) was calculated based on the formula: fasting insulin (UI/L) × fasting glucose (mg/dL)/405 derived from the computer algorithm described by Wallace et al. (8). A value of the HOMA-IR higher than 2.31 was considered for insulin resistance (9).

The inflammation status was evaluated with the high-sensitivity C-reactive protein (hsCRP) and interleukin 6. Uric acid was also measured, as part of the inflammatory profile.

### Statistical analysis

Statistical tests (distribution, correlation, and regression analyses) were performed using an SPSS (2016) software v6. The data are expressed as means, standard deviations, and medians. According to the normality test, the Pearson or Kendall correlation between variables was computed, and differences between groups were tested with ANOVA or with nonparametric Mann-Whitney U test. In the first step of the analysis, we calculated the correlation between variables. In the next step, we verified the relation between LAR and AIP in a regression model. To highlight a correlation of at least 0.300 between AIP and LAR, a power of 80%, and a two-sided alpha error of 0.05, we calculated a sample size of 80 patients using the WINPEPI version 11.65 (JH Abramson).

The final step was to assess if there were significant differences in the LAR value, according to the AIP and HOMA-IR risk categories.

## RESULTS

The population was homogenous regarding age distribution (average 24±1.54 years) with no differences in age by gender (average 24±1.21 years in men and 23±1.66 years in women; p>0.05).

The characteristics of the study group are presented in Table 1. The rate of alcohol consumption and the number of current smokers were low. The level of physical activity was below the current recommendations. Among the whole group, 21 subjects (all women) had an abnormal waist circumference, between 80 and 89 cm (average: 83.67 cm; standard deviation: 2.348); among them, five had values between 85 and 89 cm. None of the subjects had high systolic blood pressure, but four had diastolic blood pressure above 85 mmHg. No fasting abnormal glycaemia was recorded, but 18 persons had a high HOMA-IR index.

Concerning serum lipid measurements, one person had abnormal triglycerides (>150 mg/dL) and 14 had low high-density lipoprotein cholesterol values. When the AIP value was considered, 51 participants were in the low-risk range (61.4% of the total). There were 18 (21.6%) included in the high-risk and 15 (18%) in the medium-risk categories. No apolipoprotein B measurement was above the normal range values.

A strong linear relationship was found between BMI and waist circumference (r=0.7802, p=0.000035). We found a fair, significant correlation for both waist circumference and BMI with creatinine, HOMA-IR, and AIP. Adiponectin was negatively correlated to BMI, but leptin was correlated to neither BMI nor waist circumference (Table 2).

LAR was significantly correlated with the waist circumference (r=0.28577, p=0.005), LAP (r=0.31902, p=0.001), AIP (r=0.18560, p=0.05), and HOMA-IR (r=0.21588, p=0.03). In a regression model, LAR was the only predictor of AIP, after correction from smoking, alcohol, and physical activity level; this association was not attenuated by HOMA-IR, creatinine, uric acid, interleukin 6, hsCRP levels, or gender (Table 3).

**Figure 1:**
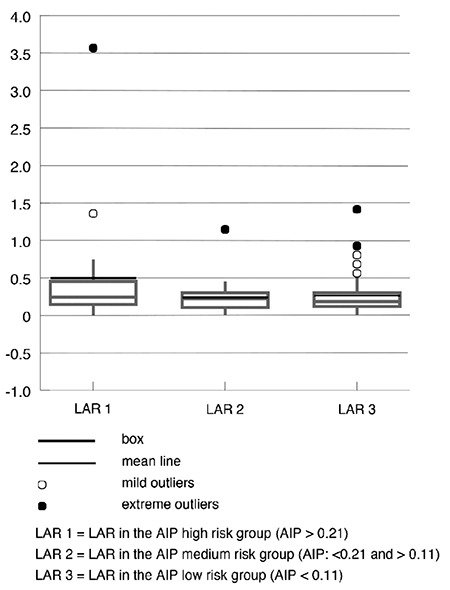
Distribution of LAR according to the AIP risk classification (p<0.05). LAR 1= LAR in the AIP high-risk group (AIP >0.21); LAR 2= LAR in the AIP medium-risk group (AIP 0.11-0.21); LAR 3= LAR in the AIP low-risk group (AIP <0.11). AIP: atherogenic index of plasma; LAR: leptin-to-adiponectin ratio

According to the AIP, the highest number of study participants (55) were in the low-risk category, while 18 were in the medium and 20 in the high risks. Inside these three AIP risk categories, the distribution of LAR was significantly different (Figure 1).

**Figure 2:**
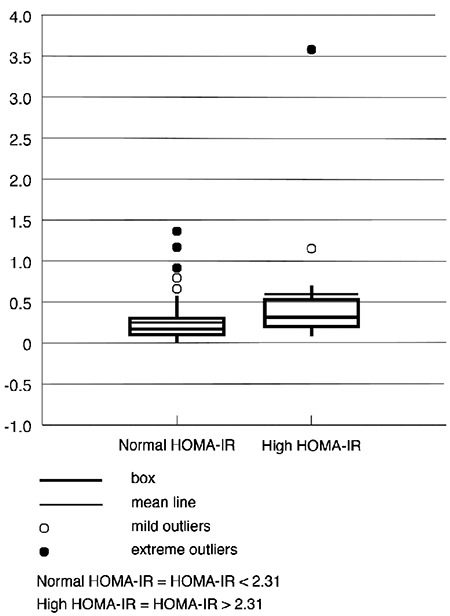
Distribution of LAR according to the (HOMA-IR; p<0.05). A HOMA-IR value of <2.31 is considered normal, whereas >2.31 is high. HOMA-IR: homeostatic model assessment of insulin resistance; LAR: leptin-to-adiponectin ratio

There were 14 subjects with a high HOMA-IR index. Comparison between low- and high-level HOMA-IR showed no distribution differences in gender (chi-squared test; p=0.35) but significant differences in BMI (p=0.005), waist circumference (p=0.001), AIP (p=0.014), and LAR (p=0.012; Figure 2). The best regression model maintained the waist circumference and hsCRP as influencers of the HOMA-IR.

LAP was significantly correlated with creatinine (r=0.22, p=0.027), leptin (r=0.25, p=0.016), LAR (r=0.31, p=0.001), and HOMA-IR (r=0.22, p=0.033). Using the backward method for multiple regression, LAR was the best correlation factor for the LAP. LAR in the highest LAP quartile was significantly higher than the rest of the group (p=0.032).

We did not find a significant correlation between the inflammatory markers (hsCRP and interleukin 6) and the adipokine profile (Table 4).

## DISCUSSION

We report in a healthy, young, and non-obese adult population a direct correlation between the unfavorable plasma adipokine profile, estimated by the LAR and the AIP, HOMA-IR, and the LAP. The link between dyslipidemia, adipokine secretion, and HOMA-IR in a population not commonly included in the cardiometabolic screening program raises questions about the age commencement of screening and the relevant tests included in the screening package. In the regression models derived from our data, LAR was best correlated with all these unfavorable biomarkers.

**Figure 3:**
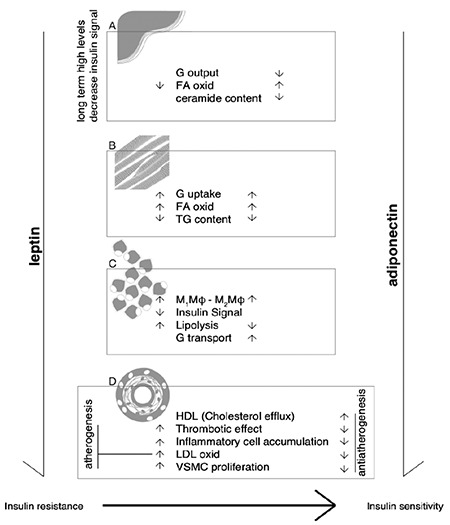
a-d. Adiponectin and leptin influences on insulin sensitivity and vascular disease. Adiponectin reduces the hepatic G output and enhances the activity of ceramidase, lowering the ceramide content in the liver cells. The activation of AMPK leads to the inhibition of lipogenesis and FA oxidation. The global activity of adiponectin is to increase insulin sensitivity. Leptin also activates the AMPK; but under long-term, high levels of stimulation, leptin decreases insulin signal, most probably through its central action (a). Leptin and adiponectin have common effects on skeletal muscle: they increase GLUT4 and the FAs oxidation and they decrease the triglyceride content (b). Adiponectin and leptin have opposite effects in adipose tissue: adiponectin has anti-inflammatory effects (M2Mφ polarization), whereas leptin increases the M1Mφ number (M1Mφ polarization). Lipolysis is increased by leptin and inhibited by adiponectin. Glucose transport via GLUT4 is increased by adiponectin and inhibited by leptin. Through these actions, adiponectin contributes to insulin sensitivity maintenance and leptin to the impairment of the insulin signal and insulin resistance (c). The pro-atherogenic effect of leptin is related to inflammatory cells accumulation, oxidative stress, VSMC proliferation, and platelet activation. Adiponectin has anti-atherogenic actions: adiponectin limits monocytes–endothelial cells adhesion, oxidative stress, VSMC proliferation, and platelet activation (d). AMPK: adenosine monophosphate-activated protein kinase; FA: fatty acid; G: glucose; GLUT4: glucose uptake through glucose transporter type 4; HDL: high-density lipoprotein; LDL: low-density lipoprotein cholesterol; TG: triglyceride; VSMC: vascular smooth muscle cells

The relationship between LAR and the metabolic and vascular risk has several pathophysiological explanations (Figure 3). Adiponectin has a large influence on lipid metabolism; this adipokine inhibits the formation of oxidized low-density lipoprotein cholesterol (10), upregulates the expression of the adenosine triphosphate-binding cassette protein 1 transporter, facilitates cholesterol efflux from macrophages to apolipoprotein A1 (11), suppresses spontaneous and catecholamine-induced lipolysis in adipocyte, and reduces the high-density lipoprotein cholesterol catabolism (12). All these, combined with the limitation of monocytes–endothelial interaction and the reduction of the oxidative stress and of the platelet activation, contribute to the anti-atherogenic effects of adiponectin. Large prospective studies have shown that high levels of adiponectin, even with weight gain, are protective for the metabolic response (13). On the other side, at the vascular level, leptin acts through common pathways with insulin (Janus kinase/signal transducers and activators of transcription and phosphoinositide-3-kinase–protein kinase B/Akt signals), extracellular signal-regulated kinase 1/2, and p38 resulting in hypertrophic effects, particularly on the smooth muscle, promoting vasoconstriction and reactive oxygen species formation (14). Leptin increases also the activity of the macrophage lipoprotein lipase, favoring the subendothelial retention of lipoproteins and stimulating the generation of foam cells.

Concerning the glucose metabolism, adiponectin directly sensitizes hepatic and skeletal muscle cells to insulin action. Leptin has a more controversial role in normal individuals as it diminishes the hepatic glucose output and increases the peripheral glucose uptake. Long-term, high leptin levels are associated with leptin resistance and contribute to a decrease in insulin sensitivity.

In adipocyte, adiponectin and leptin have different intracellular secretion pathways (15) distinctly regulated by insulin, thyroid hormones (16), glucocorticoid (17), β-adrenergic stimulation (18), local hypoxia, or inflammatory mediators. These mechanisms do not exclude the reciprocal influence on secretion. Recent experimental data substantiate the influence of leptin on adiponectin serum concentrations (19) and provide arguments for using LAR as a synthetic indicator of the adipokine profile. The high plasma level of LAR was proposed as a secretion pattern of hypertrophic adipocytes in relation to subclinical inflammation and insulin resistance (20). Studies in middle-aged (2,21) and adolescent subjects (22) confirmed that a higher LAR relates to an increase in the number of metabolic syndrome components, whereas low levels predict the regression of metabolic syndrome (23), if lifestyle factors improve.

There is evidence of the importance of the total body fat and its distribution regarding the metabolic syndrome. We did not measure the fat mass, but in the context of a normal liver function, as in our subjects, creatinine is proportional to the muscle mass and constitutes an acceptable surrogate for the fat-free mass. We can exclude circumstances that would influence the level of plasma creatinine. The influence of exogenous intake was avoided by the 10 h fasting before sample collection, none of the subjects had diabetes or other ketosis precipitating factors. The level of lipids was not high enough to interfere with the Jaffe reaction in any of our study participants.

The LAR–AIP relation was significant, and it was not influenced by the HOMA-IR, inflammatory markers (interleukin 6 and hsCRP), or anthropometric measures. While dyslipidemia is common with excessive adipose tissue, up to 10-18% of the normal BMI population has an impaired lipid profile. In our study, 21.6% of the participants were included in the high atherosclerosis risk, according to their AIP value. The anthropometric measures directly correlated with AIP, HOMA-IR, adiponectin, and uric acid suggest a gradual change toward an unfavorable metabolic profile as body fat accumulates, even before the threshold for obesity by the BMI measurement is reached. This may be argued also by the significant, direct correlation between LAR and the lipid accumulation index. As obesity and cardiometabolic risk can segregate independently, we introduced all the variables in the regression model. LAR maintained the most significant relationship.

Similar to our results, in lean subjects, plasma levels of adiponectin were inversely correlated with BMI, with no association with other parameters of adiposity, insulin sensitivity, or interleukin 8, interleukin 6, and tumor necrosis factor alpha circulating levels (24). Even if adiponectin was correlated to HOMA-IR, in the regression model, the association with LAR was stronger.

We also found a significantly higher LAR in the insulin-resistant group. This result was previously reported in older populations, where LAR was even considered a differentiator between resistant and non-resistant insulin subjects, or an insulin resistance predictor (25) LAR was directly correlated with the glucose infusion rate in the euglycemic clamp test (26) and used as an index for the treatment response in diabetes. In our study, the high HOMA-IR group had significantly higher anthropometric measures, AIP, and LAR. The link between HOMA-IR and AIP is not surprising, as glycemic control and lipid metabolism dysfunction are interconnected in the atherosclerosis pathogenesis. In patients with type 2 diabetes, AIP was strongly associated with markers of impaired glucose metabolism (27).

The involvement in insulin resistance of the high leptin and low adiponectin levels benefits from extensive experimental research; adiponectin reduces hepatic glucose output and stimulates glucose utilization in muscles. Constant high leptin levels chronically activate the Janus kinase/signal transducers and activators of transcription 3 pathway in the central nervous system and lead to leptin resistance. These experimental data provide pathophysiological arguments for the LAR–HOMA-IR correlation and a discriminative role of LAR between high and low HOMA-IR values.

Our results underline a link between the adipokine profile and LAP. This index has been used in large cross-sectional studies (7) and found to be a better predictor for diabetes than BMI in the middle-aged and old populations, in line with the hypothesis that the transition to metabolic impairment is related to fat mass accumulation (7,14). When applied to a normal BMI population, the LAP was an independent predictor of the cardiovascular risk (28). Our results confirmed the correlation between LAP, anthropometric measures and HOMA-IR, hsCRP but identified a stronger relation with the LAR. The LAP is calculated based on the waist circumference and plasma triglycerides levels; therefore, the direct link between LAR and LAP could also be the expression of the predominant unhealthy visceral fat with an abnormal secretion profile in the non-obese subjects.

We did not find a significant correlation between the inflammatory markers (hsCRP and interleukin 6) and the adipokine profile. In normal-weight subjects, the adjusted mean of the HOMA-IR was not significantly different according to the hsCRP categories; the explanation given is that, even if inflammation has deleterious vascular and metabolic effects, the relationship is not linear (29). In normal-weight subjects, a high level of interleukin 6 is associated with insulin sensitivity, although as fat mass increases, interleukin 6 contributes to insulin resistance. In normal-weight subjects, the adjusted mean of the HOMA-IR was not significantly different according to the hsCRP categories (29). We consider that our results are in the same line with these data.

In contrast with our results, in a Taiwanese population, LAR was a predictor for both inflammation and insulin resistance (30). Besides the possible racial specificities supported by other studies (2), the older age of the participants could contribute to this difference.

The strengths of our study lie in our selection of a homogenous, young population and the strict enrollment criteria that excluded major metabolic syndrome components and influencers. Accessible, widely spread biological tests were used; therefore, good candidates were gathered for screening. To the best of our knowledge, this is the first study centered on the value of LAR in cardiometabolic risk assessment, by comparing the adipokine secretion index with different other risk indicators.

There are also some limitations to our study. The number of cases is not sufficient enough for a final conclusion. A potential bias could be the predominance of the female population that makes our study more representative for the young women than for men, and the participation of the volunteers, which are generally healthier and have a healthier lifestyle than the general population (e.g. smoking and alcohol habits). We are also aware that our study cannot provide causation arguments because of its cross-sectional design.

Identifying the best suitable model for cardiometabolic risk in the young population remains a challenge for prevention medicine. Our study provides arguments that a high LAR could be a risk indicator in the young non-obese population, as it fairly correlates with other risk indicators such as plasma lipid profile, visceral fat mass distribution, and insulin sensitivity.

Longitudinal studies on cardiovascular risk are required to clarify the clinical significance of the abnormal endocrine function of the adipose tissue. These studies should take into account the predictive value of LAR for cardiometabolic diseases in the young, normal-weight population.

## Figures and Tables

**Table 1:**
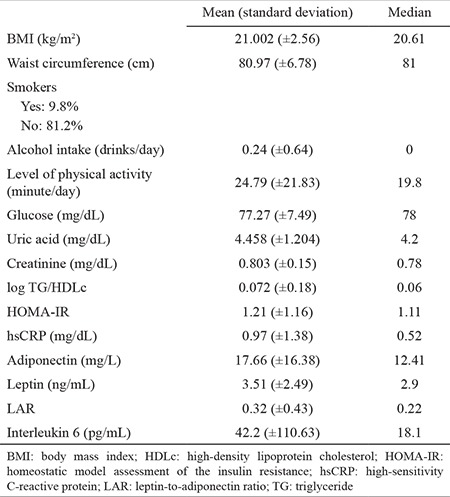
Description of the study population

**Table 2:**
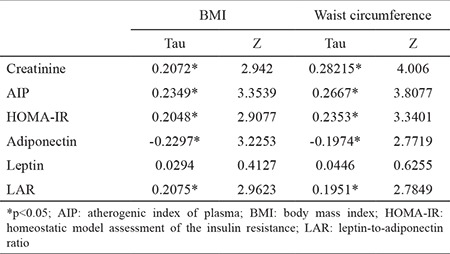
Correlation between anthropometric and biological measurements

**Table 3:**
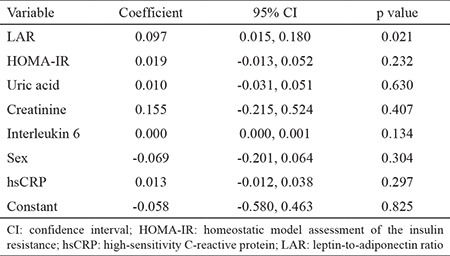
Regression model for the atherogenic index of plasma

**Table 4:**
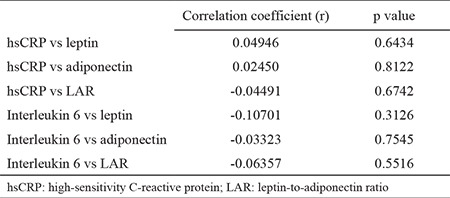
Correlation between adipokines and inflammatory markers
